# Enhancing Wholemeal Bread Shelf Life Using Optimized Mixtures of Cinnamon, Clove, and Bay Leaf Essential Oils

**DOI:** 10.1002/cbdv.202501988

**Published:** 2025-10-30

**Authors:** Mariana Pereira Silveira, Karine Guimarães Moreira, Míriam Aparecida de Aguilar Santos, Irene Andressa, Mateus Alves Araújo, Nathalia de Andrade Neves, Franciele Maria Pelissari, Marcio Schmiele

**Affiliations:** ^1^ Institute of Science and Technology Federal University of Jequitinhonha and Mucuri Valleys Diamantina Brazil; ^2^ School of Food Engineering University of Campinas Campinas Brazil; ^3^ Department of Food Science Federal University of Lavras Lavras Brazil; ^4^ Department of Food Technology Federal University of Viçosa Viçosa Brazil

**Keywords:** baking, essential oils, food preservation, fungal spoilage, natural antimicrobials

## Abstract

This study evaluated the preservative potential of essential oils (EOs) in bakery products by identifying fungal contaminants in bread, testing the in vitro antifungal activity of a mixture of cinnamon, clove, and bay leaf EOs, and assessing their effectiveness in extending shelf life. Fungal identification revealed the presence of *Penicillium*, *Aspergillus*, and *Rhizopus* species. A central composite design was used to determine the optimal EO proportions for inhibiting fungal growth. The best combination totaled 11.77 µL, consisting of 34.2% cinnamon, 42.5% clove, and 23.3% bay leaf. The minimum inhibitory and lethal concentrations of the optimized mixture were both 20 µL/mL of fungal inoculum. The application of this mixture extended the shelf life by up to 7 days when added to the dough and up to 22 days when applied to the bread surface. These results suggest that this EO combination is an effective natural antifungal agent with the potential to reduce the use of synthetic preservatives in bakery products.

## Graphical abstract

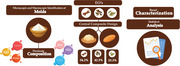

## 1 Introduction

Bakery products are characterized by their limited shelf‐life lasting 3–5 days at room temperature when no preservatives are added [1], being highly susceptible to physical, chemical, and microbiological deterioration, primarily caused by molds, yeasts, and, less frequently, bacteria [2]. Fungal contamination of food products constitutes a critical food safety concern within the food industry, as it not only results in substantial economic losses but also poses significant risks to human health [3]. The application of chemical preservatives remains the most widely adopted preservation strategy; however, prolonged use has been associated with potential adverse effects on human health [3]

The implementation of active and intelligent packaging has emerged as an effective strategy to mitigate food losses. Beyond preserving the physical integrity of products throughout the supply chain, these packaging systems contribute to maintaining the chemical and microbiological quality of foods through their antifungal, antimicrobial, and antioxidant activities [4, 5]. Consequently, in recent years, there has been growing scientific interest in enhancing the functional performance of packaging materials [6]. Essential oils (EOs) are secondary plant metabolites composed of terpenic hydrocarbons, terpenic alcohols, aldehydes, ketones, phenols, esters, ethers, oxides, peroxides, furans, organic acids, and lactones. Recently, numerous plant EOs, such as oregano, clove, basil, cinnamon, thyme, peppermint, rosemary, citrus, tea tree, and ginger, or just they active principle, as eugenol, carvacrol, thymol, menthol, muscimol, carvone, cinnamaldehyde (CMD), citral, and turmericene, has demonstrated food preservative capabilities and are used as food preservative [7]. Although the food industry primarily uses EOs as flavoring agents, they represent a natural alternative source of antimicrobial and antioxidant compounds, in addition to being recognized as safe for human consumption at low concentrations, typically below 0.1%–0.5% of the final product, depending on the type of oil and the food matrix [8, 9]. Their antimicrobial mechanism of action is based on the hydrophobic nature of EOs, which allows interaction with cell membrane lipids, thereby interfering with permeability and causing structural alterations [9].

**FIGURE 1:**
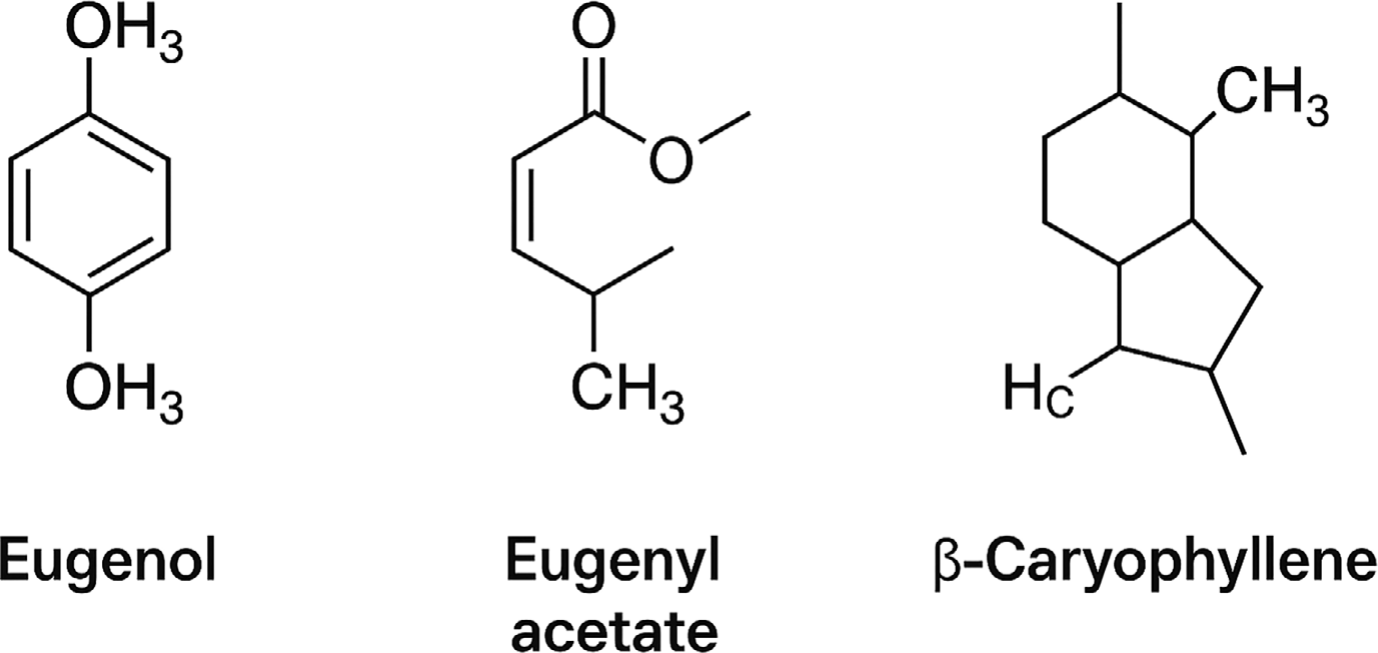
Major compounds present in clove (*Eugenia caryophyllus*) essential oil.

Clove (*Eugenia caryophyllus*) is a fragrant, dried, unopened flower bud from a perennial tree 10–20 m tall. Phytochemical studies indicate the presence of many effective compounds, including eugenol, eugenyl acetate, and β‐caryophyllene (Figure 1), with eugenol being the major bioactive compound [10]. Eugenol accounts for 77%–95% of this EO and is recognized for its biological properties, such as bactericidal, antifungal, larvicidal, antioxidant, and anti‐inflammatory, among others [11].

**FIGURE 2:**
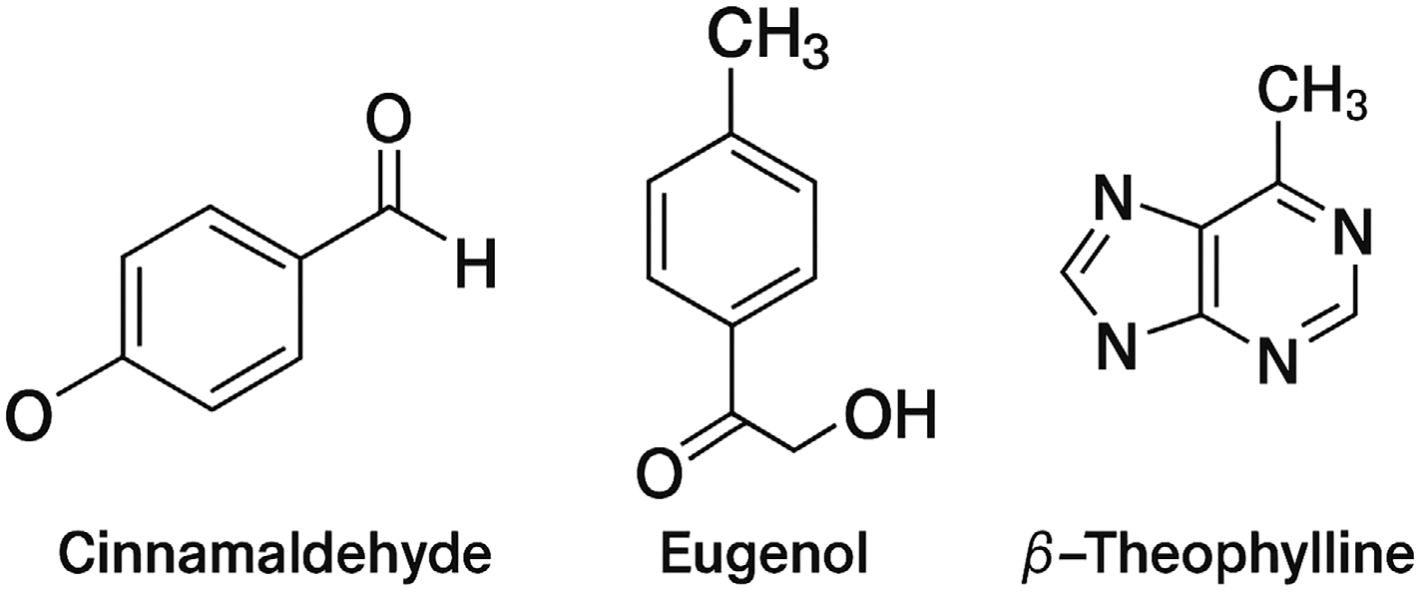
Major compounds present in cinnamon (*Cinnamomum* spp.) essential oil.

Cinnamaldehyde, eugenol, and β‐theophylline are the main active compounds of cinnamon EO (*Cinnamomum* spp.) (Figure 2). The compound has been approved by the Food and Drug Administration (FDA) [9] and has been widely used in chewing gums, ice creams, candies, beverages, and breads [7]. Cinnamon EO possesses high medicinal value due to its insecticidal, antidiabetic, antioxidant, and anti‐inflammatory properties. It also exhibits significant inhibitory effects against most foodborne microorganisms and, as a preservative, can delay spoilage in a wide range of foods, including fruits and meats [12, 13, 14].

**FIGURE 3:**
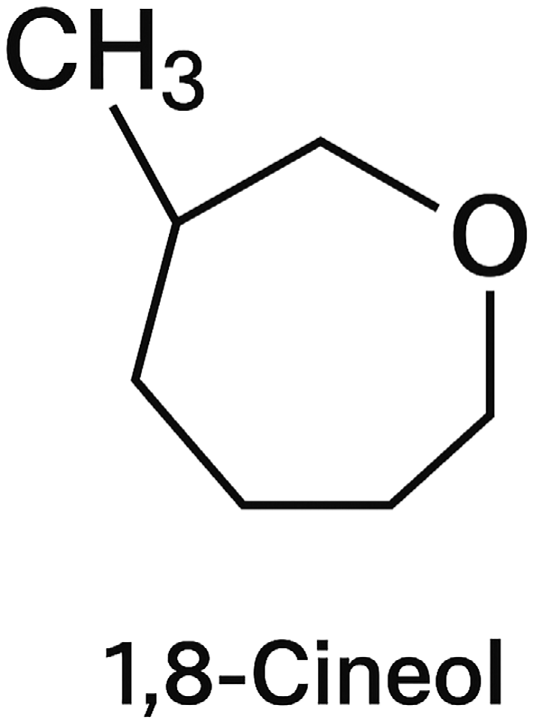
Major compound present in bay leaf (*Laurus nobilis*) essential oil.

Bay leaf (*Laurus nobilis*) is a condiment widely appreciated in traditional Brazilian cuisine. Its leaves contain approximately 1.5%–2% volatile oil, with 1,8‐cineole as the main component (Figure 3). Therefore, dehydrated leaves and bay leaf EO are employed as condiments and flavoring agents in culinary practices and the food industry [15]. Bay leaf EO exhibits antimicrobial, antioxidant, antihyperlipidemic, and antitumor activities [16, 17, 18, 19].

The application of EOs in foods represents a natural and effective strategy to combat problems related to fungal growth and is generally well accepted by consumers. However, the antimicrobial activity of EOs is closely linked to their concentration. Since high concentrations in foods may negatively affect taste and aroma, the synergistic effects of combined oils provide a promising approach to enhance antimicrobial activity while reducing the required dosage [4].

In bakery applications, EOs can be incorporated directly into the dough or applied to the surface by spraying [9]. In the first approach, EOs are uniformly dispersed throughout the product matrix, improving preservation during shelf life. However, the baking process may lead to partial thermal degradation of bioactive compounds, potentially reducing their antimicrobial efficacy [20]. In contrast, surface spraying is applied to the finished product after baking, aiming primarily to inhibit the growth of spoilage microorganisms, particularly molds, thereby contributing to shelf‐life extension [21].

Based on this context, the present study aimed to isolate and identify fungi in wholemeal bread, evaluate the in vitro antifungal activity of combined EOs of cinnamon, clove, and bay leaf, and assess the effects of adding an optimized mixture to enhance antifungal activity in wholemeal bread preservation. Both direct dough incorporation and surface spraying techniques were investigated.

## 2 Results and Discussion

### 2.1 Proximate Composition of Whole Wheat Flour

The wheat flour used in the formulations exhibited 62.92 ± 0.94% digestible carbohydrates, 1.05 ± 0.03% ash, 11.97 ± 0.60% moisture, 3.60 ± 0.06% lipids, 12.45 ± 0.20% proteins, and 8.02 ± 0.14% total dietary fiber (on a wet basis).

### 2.2 Microscopic and Macroscopic Identification of Molds

**FIGURE 4:**
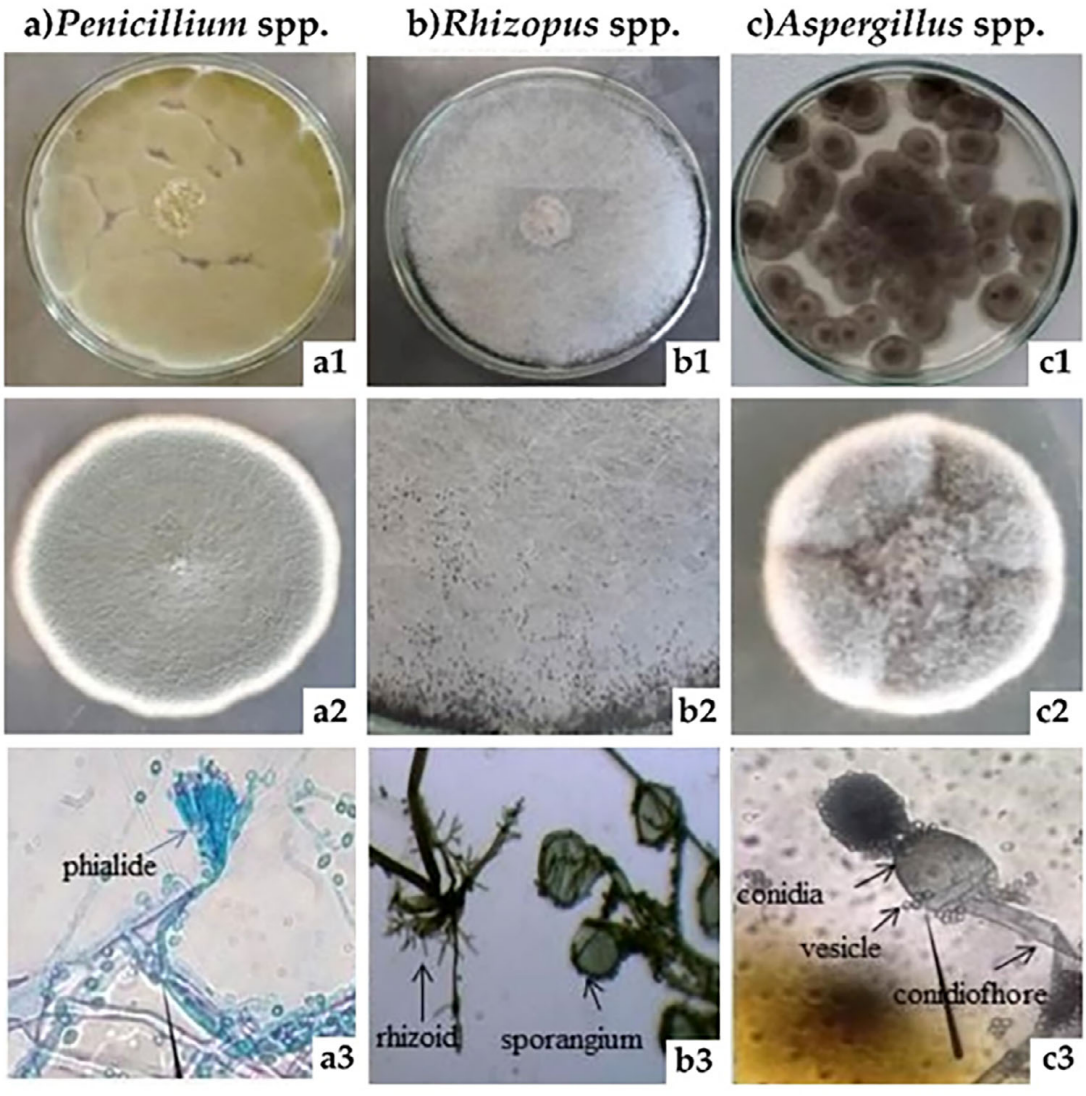
Macroscopic and microscopic aspects of filamentous fungi. (a1–a3) *Penicillium spp*.; (b1–b3) *Rhizopus spp*.; (c1–c3) *Aspergillus spp*. 1: colony on plate; 2: colony detail; 3: microscopic structures.

In the present study, three fungal **genera** were identified: *Penicillium* sp., *Rhizopus* sp., and *Aspergillus* sp., as shown in Figure 4. These microorganisms are considered natural contaminants of cereal‐based products. They are typically associated with raw materials, especially wheat flour, and are therefore commonly detected in bread and other bakery products [22, 23, 24]. Seiler [25] described colonies as greenish, with flat and slow growth, which is characteristic of the genus *Penicillium*. The conidia are spherical, unicellular, hyaline, and may be smooth or rough depending on the species. The genus *Penicillium* presents conidiophores and branches called phialides [26, 27]. These structures can be observed at the indicated points in Figure 4a, suggesting that the fungus under discussion belongs to this genus and is referred to as *Penicillium sp*.

Regarding the identification of microorganisms, *Rhizopus* is a mold that develops in tropical and subtropical regions on almost every type of food, fresh, moist, or partially dried. The most commonly found species is *Rhizopus stolonifer*. Morphologically, it presents non‐septate hyphae and cottony mycelium and produces sporangiophores in nodules, where the rhizomes are found. Usually, its sporangia are very large and black, with hemispherical columellas. The major characteristic of *Rhizopus stolonifer* is excessive growth at 25°C, and the sporangium is white at first, then becomes black with maturation [28]. The fungus isolated from the loaves (Figure 4b) presented a fuzzy appearance, white mycelium, and black sporangia; such characteristics classified the isolate as *Rhizopus* sp.

Species of the genus *Aspergillus* typically present septate conidiophores and bases shaped as “L” or “T,” commonly called foot cells, attached to vegetative hyphae. The conidiophore extends from the foot cell and may extend for a few millimeters until it reaches the vesicle [29]. The members of the genus *Aspergillus* have a green to black coloration, and *Aspergillus niger* presents a black cottony appearance, with spore heads often clearly visible [30]. As shown in Figure 4c, the features presented are similar to the description of the filamentous fungi of this genus, which allows us to infer those colonies as *Aspergillus* sp.

### 2.3 Analysis of the Experimental Design

According to the characteristics described, the agar diffusion test observed the predominance of filamentous fungi of the genus *Penicillium*, with a gradual reduction of inhibition halos throughout the incubation period for all assays (Table S1 in Suplementary Material S1).

At 1 day of incubation, the diameter of the inhibition zone ranged from 1.9 to 3 cm, and it was not influenced by the independent variables within the studied range (R^2^ = 0.6932). After 2 days of incubation, the inhibition halos showed diameters that ranged from 1.9 to 2.6 cm. According to the one‐way analysis of variance, the R^2^ value obtained was 0.8174. The clove EO showed a higher antifungal effect in the linear term (*β*_2_ = 0.16). The interactions between cinnamon and clove EOs revealed an antagonistic effect (*β*_12_ = −0.05), generating smaller inhibition halos. The interaction between cinnamon and bay laurel EOs presented a synergistic effect (*β*_13_ = 0.12), decreasing mold growth.

**FIGURE 5:**
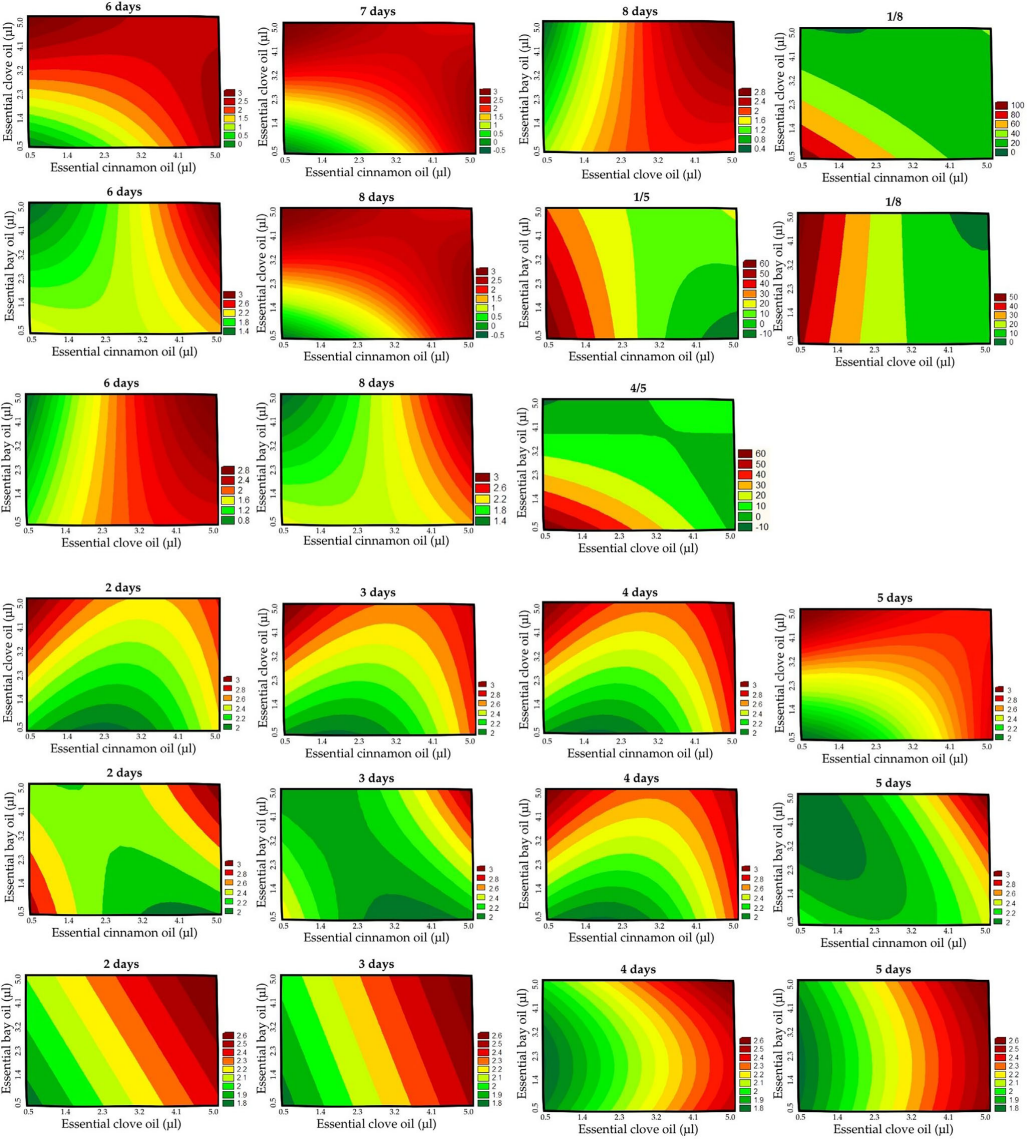
Contour curves for the evaluation of the effect of essential oils on inhibition zones on 2, 3, 4, 5, 6, 7, and 8 days of incubation, and for the percentage decrease of the halos from the first to the fifth, from the fourth to the fifth, and from the first to the eighth day.

On days 3 to 8 of incubation, the results showed an analysis of variance with R^2^ above 0.8. Between the first and the fifth day, the ANOVA showed R^2^ de 0.8670. However, between the fourth and fifth days, the R^2^ value was 0.8633; between the first and eighth days, the R^2^ value was 0.8245. In all situations, the Fcalc was higher than Ftab and p‐value <0.001, enabling the generation of contour plots (Figure 5).

The best results were the isolated factors and/or combined, in which the reduction of halos was minimal, indicating a prolonged antimicrobial effect and higher efficiency. In this case, the positive effect on the independent variables and synergistic effects were observed for negative regression coefficients. By the fifth day of incubation, the cinnamon (*β*_1_ = −7.5166) and clove (*β*_2_ = −11.9308) EOs showed a greater effect, positively influencing the maintenance of antifungal effectiveness.

Overall, the decrease in halo diameter between days 1–8 was considered of greater significance because it was the maximum incubation time evaluated. In this way, the best effect was obtained using clove EO, followed by cinnamon EO. When used alone, bay EO did not appear to have promising effects, but its combined use with clove EO was advantageous.

According to Mishra et al. [31], synergistic effects are due to the presence of phenolic and alcoholic compounds in EOs and the similarity between the phenolic compounds. The interaction between non‐oxygenated and oxygenated monoterpene hydrocarbons contributes to the antagonistic effect of EOs. The most commonly accepted mechanism for synergistic inhibition activity is sequential inhibition of a common biochemical pathway, inhibition of protective enzymes, and the use of active agents in the cell wall to enhance the uptake of other antimicrobials.

### 2.4 Validation of Mathematics Models

The validation of the mathematical models (Table S2 in Supplementary Material S1) for predicted values in the statistical analysis (Table 1) showed the real values were not consistent with those predicted by mathematical models in most cases (relative deviation >20%). However, by the sixth to seventh day of incubation, the diameter of the inhibition halos exhibited the expected results. Two solutions were evaluated for validation of the mathematical models (solutions 1 and 2) with different proportions of the EOs studied. The results identified Solution 1 as ideal, as the essays presented an inhibition halo higher than the diameter predicted by the mathematical model and showed a lower relative deviation in most cases compared with Solution 2.

**TABLE 1:** Limitations used to define the optimum point.

Variable	Purpose	Minimum limit	Maximum limit	Importance	Solution 1	Experimental value	Relative deviation (%)	Solution 2	Experimental value	Relative deviation (%)
X_1_	Maximize	−1.68	1.68	5	1.33^a^	—	—	1.06^a^	—	—
X_2_	Maximize	−1.68	1.68	5	1.68^a^	—	—	0.44 ^a^	—	—
X_3_	Maximize	−1.68	1.68	2	0.37^a^	—	—	1.68^a^	—	—
Day 2	Maximize	1.90	2.60	5	2.56^b^	3.90	34.40	2.47^b^	3.80	35.00
Day 3	Maximize	1.90	2.60	5	2.64^b^	3.80	30.50	2.67^b^	3.70	27.84
Day 4	Maximize	1.80	2.60	5	2.64^b^	3.60	26.70	2.74^b^	3.50	21.71
Day 5	Maximize	1.20	2.60	5	2.41^b^	3.40	29.10	2.58^b^	3.30	21.82
Day 6	Maximize	0.60	2.60	5	2.44^b^	3.00	18.70	2.61^b^	3.00	13.00
Day 7	Maximize	0.60	2.60	5	2.35^b^	2.80	16.10	2.24^b^	2.70	17.04
Day 8	Maximize	0.50	2.60	5	2.39^b^	2.50	4.40	2.63^b^	2.30	14.35
1/5	Minimize	0	60	5	8.40^c^	5.98	49.60	0.33^c^	34.29	99.04
4/5	In range	0	42.9	3	9.22^c^	3.12	44.20	3.13^c^	5.71	45.18
1/8	In range	3.70	76.19	3	18.08^c^	35.98	49.60	1.11^c^	5.71	80.56

X_1_: cinnamon essential oil; X_2_: clove essential oil; X_3_: bay laurel essential oil;

^a^ Coded level to optimize the concentration of essential oils;

^b^ Optimized diameter of inhibition halos;

^c^ Optimized percentage decrease of inhibition halo.

Moreover, a lower inoculum concentration (1.9 x 10^6^ CFU/ g) could justify the larger halos. In *in vitro* assays, the incubation period for mold counts extends from 5 to 7 days. Therefore, the model is applied to the average time required for colony development, which makes this methodology efficient for assessing the inhibitory effect of antifungal agents.

In vitro, the antifungal effect of EOs varied according to their composition, concentration, and the classification of the food spoilage fungi. In previous works, clove EO has shown the ability to inhibit the growth of *V. inaequalis*, *C. albican*s, *C. glabrata*, and *C. tropicalis* [32]. The clove EO exhibited the highest inhibitory effect among the EOs tested in the present study, while the inhibition of *Penicillium* spp. was lower when compared to the other genera isolated from the loaves. Xing et al. [33] determined the antifungal activity of cinnamon EO against *Rhizopus nigricans*, *Aspergillus flavus*, and *Penicillium expansum*. The results showed that the minimum inhibitory concentrations (MICs) for those fungi were 0.64% (v/v), 0.16% (v/v), and 0.16% (v/v), respectively. Therefore, the best effect was observed for the inhibition of *R. nigricans*. Such outcomes may be associated with the predominance of *Penicillium* spp. in the study and its probable resistance to the inhibitory effects of eugenol and linalool.

Table 1 shows the antifungal effect optimization (considering the dependent variables statistically significant), the desired purpose, and the variables' importance. The analysis resulted in an optimal solution with a desirability of 93.8%, which corresponds to 11.77 µL from the blend of EOs: 34.2% cinnamon EO, 42.5% clove EO, and 23.3% bay laurel EO.

### 2.5 MIC and Minimal Lethal Concentration

The inoculum of the total colonies developed in the loaves presented both MIC and minimum lethal concentration (MLC) at 20 µL of the EOs blend per mL of inoculum (1.9 x 10^6^ CFU/ g). For the fungi in isolated form, the MICs were 5, 10, and 5 µg/mL against *Aspergillus* sp., *Penicillium* sp., and *Rhizopus* sp., respectively. Meanwhile, the MLCs were 10, 20, and 10 µL of the EOs blend per mL of inoculum. Thus, due to higher required concentrations, the genus *Penicillium* sp. was identified as having higher resistance to the EOs blend compared to the other fungi identified in whole‐grain bread.

The interaction between the EOs components may have affected their individual form of action due to the synergism and antagonism presented. The results of the present study demonstrated satisfactory inhibition of fungal growth, which leads to deducing the optimized blend of EOs, which may decrease fungal development in whole‐grain bread.

### 2.6 Application of EOs in the Dough

**FIGURE 6:**
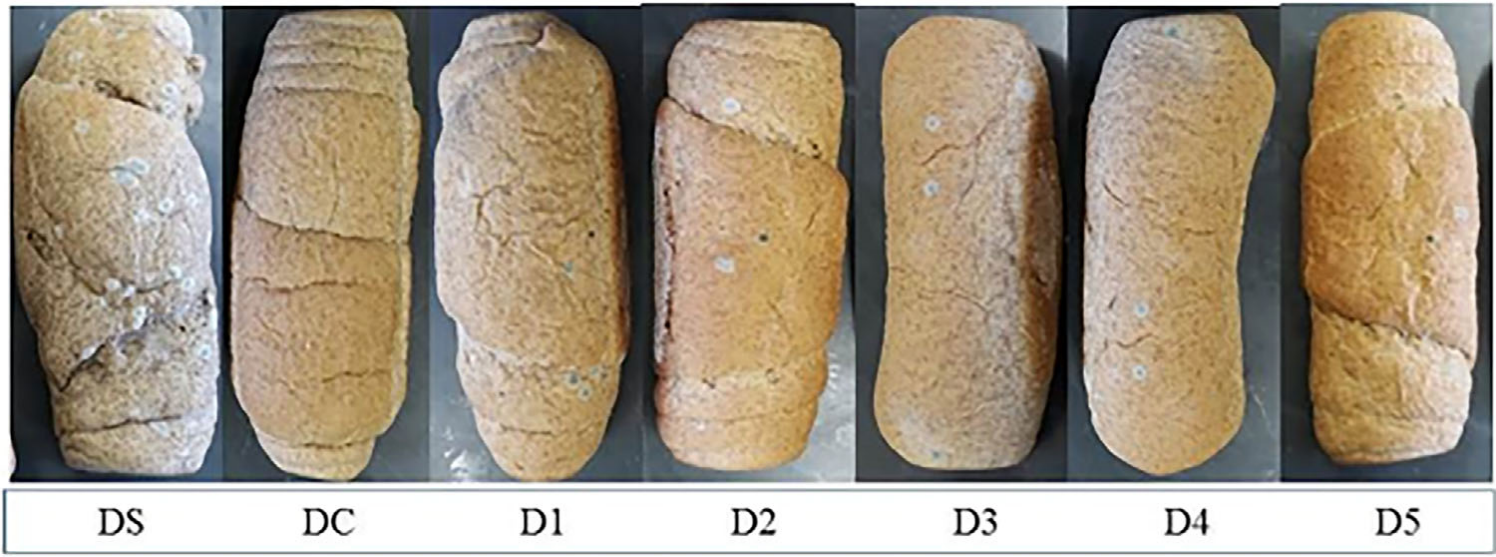
External appearance of whole wheat breads with cinnamon, clove, and bay essential oils after 7 days of storage. DS—positive control (without the addition of preservative), DC—negative control (with commercial preservative based on calcium propionate), and different concentrations of EOs (D1: 0.28 µL, D2: 0.57 µL, D3: 0.85 µL, D4: 1.13 µL, and D5: 1.42 µL of EO/g of dough).

No fungal growth was detected until the fourth day of storage. However, on the seventh day, all samples showed visible fungal development, with the sample without any preservative (DS) significantly different from the others, with a higher number of CFU/g (3.07 ± 0.34 x 10^2^). Under these conditions, fungal colonies were already visible in the product. Visual analysis showed that the sample without any preservative exhibited more prominent colonies, while the others showed fewer and more isolated ones, as evidenced in Figure 6. Nevertheless, it can be considered that none of the samples were suitable for consumption.

Baking bread presents a challenge for maintaining the biological activity of EOs, as high temperatures can lead to volatilization and degradation of their constituents. However, studies indicate that this loss is not complete, and more thermostable compounds remain active after thermal processing. Picolloto et al. [34] observed that, among cinnamon and oregano oils, cinnamon oil exhibited greater resistance to thermal volatilization, better preserving its composition. Phenols and aromatic aldehydes, such as cinnamaldehyde (cinnamon) and eugenol (clove), showed higher stability under heat, while volatile monoterpenes were preferentially degraded. Additionally, the use of closed systems reduced thermal decomposition, highlighting the role of the environment in retaining bioactive compounds.

The presence of these phenolic compounds and aromatic aldehydes is important, as it is associated with thermal resistance and the antimicrobial activity observed in the breads. Despite exposure to baking, an initial preservative effect was still evident, with no fungal growth detected until the fourth day of storage. By the seventh day, all samples exhibited visible colonies, which were more pronounced in the bread without preservatives (DS: 3.07 ± 0.34 × 10^2^ CFU/g), while samples containing EOs showed smaller and more isolated colonies.

Previous studies reinforce the complexity of using EOs for bread preservation. The bread's shelf‐life in all treatments was more than four days but less than seven days, suggesting a partial preservative effect. The use of commercial packaging with a low oxygen barrier can enhance these effects and represents a cost‐effective strategy for consumers. Evaluating the preservative effect of thyme EO, Debonne et al. [33] concluded that, despite its promising in vitro potential, no extension of shelf‐life could be attributed to the EO. They highlighted that, to achieve effective biopreservation, the formulation should include fewer aromatic compounds and an emulsifier to promote homogeneous dispersion of the antifungal active components. These findings align with the present results, emphasizing that both the composition of the EO and the formulation method critically influence their preservative efficacy in baked products. The results of the analysis of water activity, specific volume, and instrumental color of the bread are presented in Table 2. The bread showed high water activity values (>0.95), with no statistical difference between samples. From the consumer's point of view, specific volume is an extremely relevant parameter for the quality of sandwich bread. The results showed that the volume of the control bread (DS and DC) was higher than the other treatments. A statistical difference was observed, with an increase in the concentration of EOs resulting in a decrease in specific volume. Thus, it can be inferred that the incorporation of EOs negatively influenced the yeast metabolism during fermentation, decreasing the production of CO_2_. Regarding instrumental color, none of the evaluated parameters were statistically influenced by the addition of EOs.

**TABLE 2:** Results of analysis of water activity, specific volume, and instrumental color of whole wheat bread with application of cinnamon, clove, and bay laurel essential oils to the dough.

Sample	Water activity	Specific volume (g.cm^−3^)	L*	a*	b*
DS	0.9632 ± 0.0044	4.22 ± 0.08	66.62 ± 0.34	4.52 ± 0.11	16.86 ± 0.03
DC	0.9454 ± 0.0065	4.22 ± 0.04	68.71 ± 0.13	4.15 ± 0.16	16.40 ± 0.26
D1	0.9499 ± 0.0049	3.89 ± 0.04	65.57 ± 0.46	4.13 ± 0.05	16.65 ± 0.15
D2	0.9643 ± 0.0016	3.63 ± 0.02	65.90 ± 0.13	4.42 ± 0.07	16.90 ± 0.08
D3	0.9581 ± 0.0058	3.69 ± 0.04	65.85 ± 0.18	4.44 ± 0.03	16.94 ± 0.06
D4	0.9559 ± 0.0018	3.63 ± 0.02	65.95 ± 0.98	4.39 ± 0.14	16.99 ± 0.54
D5	0.9698 ± 0.0032	3.43 ± 0.11	66.23 ± 1.54	4.59 ± 0.54	16.92 ± 0.14
p‐value	0.082	<0.001	0.097	0.144	0.091
R^2^	0.151	0.855	0.138	0.109	0.143

Mean of three repetitions ± standard deviation. L*: lightness; a*: red‐green coordinate; b*: yellow‐blue coordinate.

The moisture content of the bread significantly decreased for all samples over the storage period (Table 1). It was observed that on the first day, there was a significant difference between the samples (*p* = 0.001), with a tendency of moisture reduction as EO concentration increased. However, the linear adjustment is low (R^2^ = 0.441), indicating that the behavior of the responses is not satisfactorily justified by mathematical models. Regarding the storage period, there was a significant reduction in moisture for all treatments. This effect can be explained by the phenomena that occur in bread during aging, mainly due to the redistribution of water between the starch and protein fractions. Additionally, there is moisture migration from the crumb to the crust of the bread, and exposure to the atmosphere, a known effect as syneresis and retrogradation. Over the storage period, the hydrogen bonds of the starch chains with water are broken due to having lower activation energy than the hydrogen bonds between the hydroxyl groups of the starch chains, promoting water expulsion and starch recrystallization [35].

The results of crumb firmness analysis (Table 3) indicated a linear behavior, with a gradual reduction in softness over the storage period. The increase in firmness was expected due to subprocesses related to aging: moisture transfer from the crumb to the crust and intrinsic firmness of the starchy material, which is associated with starch recrystallization during storage.

**TABLE 3:** Results of moisture and crumb firmness analysis of whole wheat bread crumb with application of cinnamon, clove, and bay laurel essential oils to the dough.

	Moisture (%)				Crumb Firmness (N)			
Sample	Day 1	Day 4	*p*‐Value	R^2^	Day 1	Day 4	*p*‐Value	R^2^
DS	36.22 ± 0.13	30.87 ± 0.12	0.002	0.751	2.17 ± 0.11	4.86 ± 0.53	<0.001	0.840
DC	34.60 ± 0.09	33.14 ± 0.21	<0.001	0.930	2.84 ± 0.39	5.38 ± 0.68	<0.001	0.870
D1	34.97 ± 0.11	32.49 ± 0.14	<0.001	0.873	6.65 ± 0.57	16.72 ± 1.11	<0.001	0.829
D2	33.17 ± 0.16	32.55 ± 0.05	<0.001	0.953	8.96 ± 0.54	19.19 ± 1.85	<0.001	0.931
D3	32.55 ± 0.14	32.17 ± 0.22	0.005	0.687	10.67 ± 0.72	27.91 ± 2.81	<0.001	0.921
D4	33.25 ± 0.07	30.47 ± 0.11	0.004	0.701	11.41 ± 0.36	19.26 ± 1.95	<0.001	0.907
D5	34.29 ± 0.16	32.54 ± 0.03	<0.001	0.957	7.00 ± 0.39	12.90 ± 0.70	<0.001	0.910
*p*‐Value	0.001	0.828			<0.001	<0.001		
R^2^	0.441	0.002			0.575	0.336		

Mean of three replicates ± standard deviation.

Among the treatments, crumb firmness increased until the concentration of Eos reached 0.85 µL/g dough, with a subsequent decrease for higher concentrations. This behavior may be related to some type of interaction between the EOs and the formulation constituents, where high concentrations may mitigate internal moisture transfer and starch retrogradation. Additionally, some nonpolar components present in the EOs may act as crumb softeners and moisture retainers through hydrophobic interactions, reducing the rate of water migration and the syneresis effect.

### 2.7 Application of EOs on the Bread Surface

No fungal growth was detected until the fourth day of storage. However, on the seventh day, all samples showed visible fungal development, with the sample without any preservative (DS) significantly different from the others, with a higher number of CFU/g (3.07 ± 0.34 x 10^2^). Under these conditions, visually, the bread containing 3.0 µL EO/g of dough (Sample S12) showed fungal colonies only after 22 days, while the standard sample (SS) demonstrated visual growth after 6 days of storage. Corroborating with visual analyses, microbial counting (Table S3 in the Supplementary Material S1) indicated satisfactory results regarding inhibiting fungal growth by adding EOs on the surface of whole wheat bread.

Microbiologically, bread without preservative addition (SS) is suitable for consumption up to the tenth day. Samples S3, S6, and S9 remained viable until the sixteenth day of consumption, while SC and S12 showed counts below the limit until the twenty‐second day of storage. Commercial whole wheat breads typically have a shelf life of 14 to 21 days. Therefore, to preserve the bread for 2 weeks, the EO concentration used in sample 3 (0.75 µL EO/g dough) would be sufficient. However, to extend the shelf‐life further, we recommend using the maximum concentration tested in this study (3.00 µL EO/g dough). Statistically, there was no difference between samples S3 and S6 after 10 and 13 days of storage, but both differed from SS, which showed a higher mold count. On the sixteenth day, a similar behavior occurred, with SC, S3, S6, and S9 showing no difference. After 19 and 22 days of storage, it was observed that for samples with higher EO concentrations (S9 and S12) and SC, there was no statistical difference. However, considering the mentioned limit, sample S9 was deemed unfit for consumption.

**FIGURE 7:**
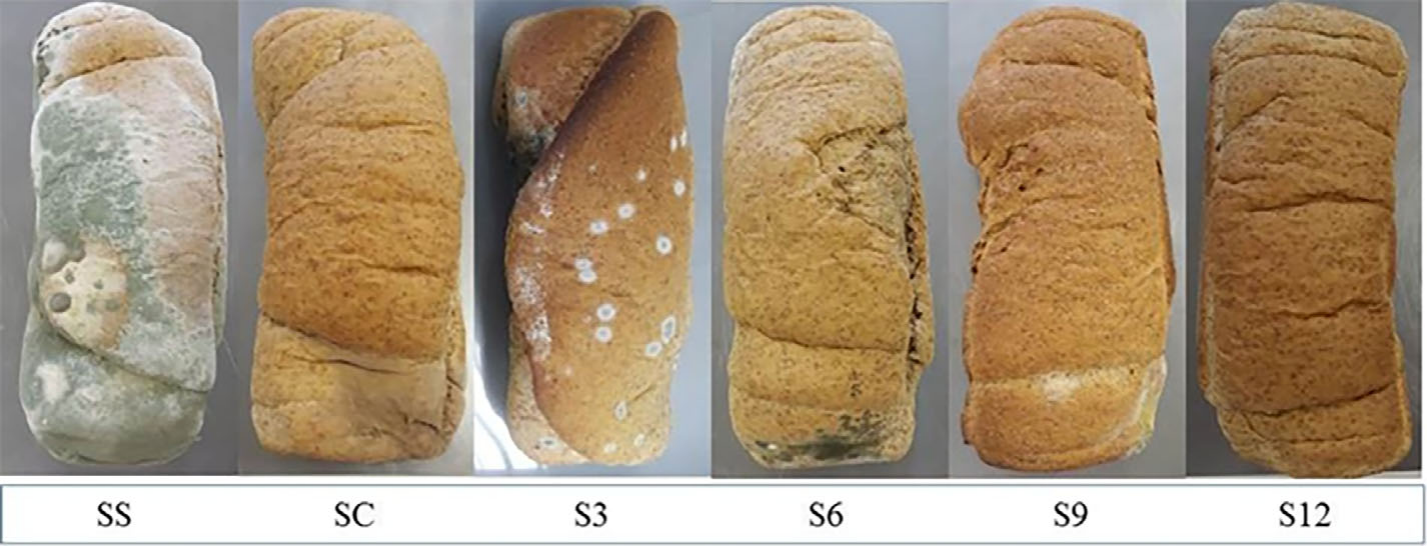
Whole wheat sandwich breads with surface application of cinnamon, clove, and bay leaf essential oils after 22 days of storage. Treatments: SS—positive control (without preservative); SC—negative control (with commercial preservative based on potassium sorbate); S3, S6, S9, and S12 – breads sprayed with essential oil mixtures at concentrations of 0.75, 1.50, 2.25, and 3.0 µL of EO/g of dough, respectively.

Figure 7 illustrates the appearance of the breads after twenty‐two days of storage. In SS and those with the lowest EO concentration (S3), advanced deterioration was observed, while in the others, smaller colonies emerged. Mold contaminations do not pose a significant risk to human health, mainly because consumers are unlikely to ingest a contaminated product, as these fungi are usually visible to the naked eye and impart a characteristic smell and taste to the food. Therefore, despite the count being below 5x10^3^ CFU/g, the breads were no longer suitable for consumption in some cases (Samples SC and S12).

It is worth noting that commercial breads typically have an average shelf‐life of 21 days, using preservatives applied both in the dough and on the surface. Therefore, this application method is an effective way to extend the product's shelf life.

The results of the crumb water activity and instrumental color analysis of the bread (Table 2) indicated no significant difference between the treatments for any of the evaluated parameters. This result was expected since there was no variation in the bread formulation, only in the preservative sprayed after baking. The whole wheat bread showed high water activity values (>0.90) and instrumental color with a characteristic hue of whole wheat bread.

The average moisture content of the bread on all analysis days was higher for samples with the addition of 3.00 µL EO/g dough (Table 3). However, only samples SC and S3 showed significant differences (p < 0.05) over time. During the aging process of bread, starch retrogradation, changes in the gluten network, and water redistribution typically occur, usually resulting in reduced moisture content [36]. The commercial preservative spray (sample SC) treatment resulted in bread maintaining higher moisture content over time. However, with the spray of EOs at the lowest concentration (0.75 µL EO/g dough), a decrease in moisture content was observed.

There was a significant difference among the samples after 4 and 16 days of storage. In both cases, it was found that increasing the concentration of EOs resulted in bread with higher moisture content. These results suggest that adding this bio‐preservative may positively influence moisture retention, favoring a product with better sensory characteristics throughout its shelf‐life. One of the factors that may have resulted in this behavior is likely related to the lipophilic characteristic of EOs. Thus, the surface layer resulting from the spray reduced the rate of water evaporation (syneresis).

However, it is important to note that the standard deviation values related to the variation of analysis replicates were relatively low, favoring statistical differences between the samples when considering the same storage time. When examining the behavior over the shelf life, a low regression coefficient was found, indicating that mathematical models could not adequately explain the experimental results.

The crumb firmness of the samples (Table 3) gradually increased over the storage period, showing significant differences for all samples. This parameter increased throughout the storage period. There was a significant difference among the samples on all days analyzed, except on the twenty‐second day. The bread exhibited lower firmness with the increase in EO concentration.

Rashedd et al. [37] found similar results, reporting a better inhibitory effect of orange peel EO in treatments with spraying on all slices of bread compared to addition to the dough, application to the surface of the entire loaf, and inside the packaging. Sensory‐wise, the ratings obtained for this treatment regarding specific volume, symmetry, crust characteristics, crumb and crust color, taste, texture, and aroma were also significantly superior to the other samples.

## 3 Conclusions

The results of this study demonstrated that EOs significantly inhibit the growth of filamentous fungi commonly found in whole wheat bread, such as species of *Penicillium*, *Rhizopus*, and *Aspergillus*. Clove EO exhibited the strongest antifungal activity, followed by cinnamon EO, while bay leaf EO showed synergistic potential when combined with the others. Overall, the incorporation of these EOs affected the specific volume and moisture content of the bread, likely due to their impact on yeast metabolism and water redistribution during storage.

Evaluation of mixtures of the three EOs allowed the determination of the optimal ratio: 34.2% cinnamon EO, 42.5% clove EO, and 23.3% bay leaf EO. At this ratio, both the MIC and the MLC against fungi cultured on the breads were 20 µL of the EO mixture per mL of inoculum.

Although the application of the EO mixture in the dough did not provide a prolonged preservative effect in whole wheat breads, spraying 3 µL EO per gram of dough on the surface resulted in breads with higher moisture content and lower firmness throughout the 22‐day shelf life. Additionally, microbiological analyses indicated a delay in fungal growth, with the most effective results observed at this concentration. However, it is important to consider that the application of EO mixtures on breads of varying shapes may require concentration adjustments. The surface contact area is a critical factor influencing the efficacy of this biopreservative, as fungal growth occurs more rapidly and is more visually noticeable to consumers on larger exposed surfaces.

Therefore, further research is warranted, focusing on sensory acceptance, formulation optimization, and packaging innovations to safely extend the shelf life of breads while preserving product quality and freshness.

## 4 Materials and Methods

Cinnamon (branch distillate), clove (leaf distillate), and bay laurel (leaf distillate) EOs were commercially obtained in sealed amber bottles (10 mL). Their application was determined based on preliminary trials assessing their effects on the technological properties and antifungal activity of powdered spices incorporated into sandwich breads.

### 4.1 Proximate Composition of Whole Wheat Flour

The proximate composition analysis of whole wheat flour was performed based on the following methods [38]: moisture content was determined by method 44‐15.02, which quantifies the water content in the sample through drying; ash content was evaluated by method 08‐12.01, which measures the inorganic minerals present in the sample after incineration; lipids were extracted using method 30‐25.01, which involves solvent extraction for fat quantification; proteins were quantified using method 46‐11.02 (*N* = 5.7), based on the Kjeldahl method, which determines protein content by measuring nitrogen. The content of digestible carbohydrates (sugars and starch) was determined using the method ISI 27‐1e [39], and the total dietary fiber content was calculated by difference.

### 4.2 Bread Preparation

The whole wheat bread was produced using the following ingredients: whole grain wheat flour (100% w/w flour basis), sodium chloride (1.8%), instant baker's yeast (1.6%), hydrogenated vegetable fat (4%), sucrose (4%), whole milk powder (4%), bread improver (a complex containing alpha‐amylase, ascorbic acid, and sodium stearoyl‐2‐lactylate) (1%), and water (55%). Bread was prepared following Nascimento et al. [40] with modifications.

Briefly, breads were produced using a modified straight dough method. Doughs were mixed and kneaded mechanically (GPaniz, AE‐15L, Caxias do Sul, Brazil), divided into portions of 400 ± 2 g, manually rounded, rested for 10 min, molded (Venancio, MPV35, Venâncio Aires, Brazil), and placed in greased aluminum pans measuring 100 mm (width) × 240 mm (length) × 50 mm (height). Subsequently, the doughs were fermented at 30 ± 3°C and 95 ± 3% relative humidity until resilience loss was detected by manual touch (Lucadema, RFE38, São José do Rio Preto, Brazil), and baked at 180 ± 5°C for 25 ± 2 min (Venax, FFI 440, Venâncio Aires, Brazil).

### 4.3 Inoculum Preparation

The breads were stored in low‐density polyethylene (LDPE) packaging at room temperature (18–22 °C) for 9 days, during which fungal colony development was monitored for subsequent inoculum preparation. The inoculum was then prepared following Ju et al. [41] with modifications. Brieflysamples were collected from the surface of whole grain loaves without preservatives, which were stored in LDPE packaging at room temperature (18–22°C) for 8 d, a period in which the visual development of fungal colonies was observed. The samples were transferred to 1% nutrient broth and incubated at 25 ± 1°C for 24 h. Dilutions were made in 1% saline solution, and 100 µL were inoculated into Petri dishes containing Potato Dextrose Agar (PDA) medium, acidified with 10% tartaric acid to reach a pH of 3.5. The plates were incubated at 25 ± 1°C for 5 d, and colony counting was performed after this period.

### 4.4 Microscopic and Macroscopic Evaluation of Molds

Both macroscopic and microscopic evaluations of the fungal colonies were performed according to Lay et al. [42], with modifications. The macroscopic characteristics evaluation was performed through image recording using a 13 MP digital camera (Samsung Galaxy J5 Pro). Visual examination of the fungal colonies was based on color, morphology, colony growth, and sporulated structures. Microscopic analysis was conducted using the Carl Zeiss Microscopy GmbH microculture method with a 100X objective lens.

### 4.5 Experimental Design

The assays to estimate the effectiveness of EOs on mold growth were evaluated by applying a Central Composite Design (CCD) method with three independent variables: X_1_‐Cinnamon EO, X_2_‐Clove EO, and X_3_‐Bay laurel EO. The method was performed according to the model presented in Equation 1, proposed by Rodrigues and Iemma [43]. The coded levels of the independent variables ranged from −1.68 to +1.68. The real concentration levels for the three independent variables varied from 0.5 (−1.68) to 5 µL (+1.68) of each EO (defined by pre‐tests).

(1) $$\begin{array}{ccc} Y & = & \beta_{0}+\beta_{1}x_{1}+\beta_{2}x_{2}+\beta_{3}x_{3}+\beta_{11}{x_{1}}^{2}+\beta_{22}{x_{1}}^{2}\\ & & +\beta_{33}{x_{3}}^{2}+\beta_{12}x_{1}x_{2}+\beta_{13}x_{1}x_{3}+\beta_{23}x_{1}x_{3} \end{array}$$

Where: Y = observed value for the dependent variable Y (inhibition zone diameter); $\beta_{0}$ = constant of regression; *β*_i_, *β*_ii_, and *β*_ij_ = coefficients of linear, quadratic, and interaction regressions, respectively; x_i_ and x_j_ = coded values of the independent variables. The response variable was antimicrobial activity, assessed through the disk diffusion method, as described by Carvalho et al. [44].

### 4.6 Agar Well Diffusion Method

The antifungal activity of the EOs was evaluated using the agar well diffusion method, according to Hayhoe and Palombo [45], with modifications. Once the medium had solidified (acidified PDA to a pH of 3.5), perforations of 10 mm diameter were made into the central portion of the plates, called wells. In each plate, 100 µL of inoculum was added at a concentration of 2 x 10^6^ CFU/g from the original microbiota in whole grain bread. Subsequently, according to the experimental design, the mixtures of EOs were applied to the wells. Experiments were performed in triplicate for each concentration condition. The plates were incubated at 25 ± 1°C for 8 days. The inhibition zone diameters were measured daily, in triplicate. Then, the optimization of the proportions of EOs was performed to validate the results and to evaluate the lowest concentrations necessary for the inhibitory and lethal effects on the selected molds.

### 4.7 MIC and MLC From the Optimized Point

The MIC and MLC were determined using the microdilution broth method in 96‐well microtiter plates. Sequentially, 100 µL of acidified PDA media, 50 µL of the inoculum (1.8x10^6^ CFU/ g), and the EOs mixture were added to the cells, with six replicates. The proportion of EOs established in the CCD optimization step was applied. The concentrations tested were 5, 10, 20, 40, 60, 80, 100, 120, 140, 160, 180, 200, 240, 280, 320, 360, and 400 µL of EOs per mL of inoculum. The MIC was defined as the lowest concentration of EOs at which no visible fungal growth was observed after 48 h, while the MLC was determined after 96 h.

### 4.8 Addition of EOs to the Dough

The treatments performed were: DS—positive control (without the addition of preservative), DC—negative control (with commercial preservative based on calcium propionate), and 5 samples with different concentrations of EOs (D1: 0.28 µL, D2: 0.57 µL, D3: 0.85 µL, D4: 1.13 µL, D5: 1.42 µL of EO/g of dough). The commercial preservative and the optimized blend of cinnamon (34.2%), clove (42.5%), and bay laurel (23.3%) EOs were added to the dough during the mixing stage of the ingredients.

**FIGURE 8:**
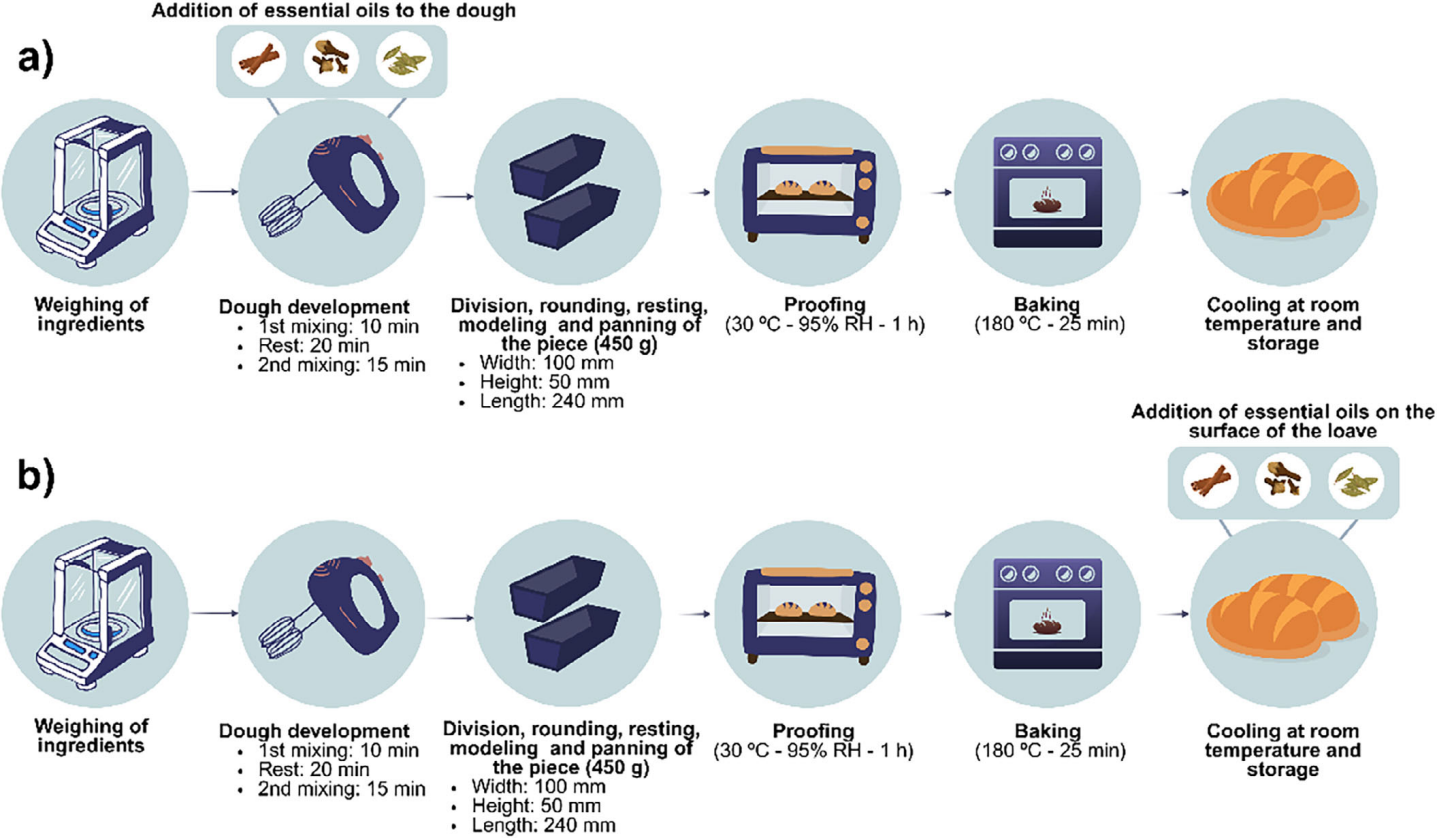
Bread preparation with the addition of essential oils to the dough (a) and to the surface (b).

The bread‐making process with the incorporation of EOs into the dough is illustrated in Figure 8b. After baking, the breads were cooled for about 90 min, packed in LDPE packaging, and stored at room temperature for 7 days. During the storage period, the daily average temperature ranged between 20.8 and 22.9°C, the daily average relative humidity between 68.2 and 76.2%, and the daily average rainfall index between 0 and 4.8 mm, according to records from the Center for Weather Forecasts and Climate Studies of INPE [46].

### 4.9 Application of EOs on the Surface

The breads were produced in a single batch and separated into groups for treatment application. For the surface spraying technique, the following assays were conducted: SS—positive control (without the addition of preservative), SC—negative control (with commercial preservative based on potassium sorbate), and 4 samples with different concentrations of EOs identified as S3, S6, S9, and S12, with spraying of 0.75, 1.50, 2.25, and 3.0 µL of EO/g of dough.

The process of bread preparation with the application of EOs on the surface is illustrated in Figure 8a. Both the commercial preservative and the EOs were previously diluted in 50 mL of food‐grade ethanol, recognized as safe for food applications. The resulting solutions were manually and evenly sprayed onto the bread surface once the loaves reached 60°C, a condition chosen to promote the evaporation of the cereal alcohol and ensure that only the active compounds of the EOs remained on the bread surface. Following application, the breads were cooled, packaged in LDPE bags, and stored at ambient temperature.

During the storage period, the daily average temperature ranged between 15.0 and 20.7°C, the daily average relative humidity between 66.4% and 96.0%, and the daily average rainfall index between 0 and 70.4 mm, according to records from the Center for Weather Forecasts and Climate Studies of INPE [46].

### 4.10 Bread Characterization

The breads were evaluated for water activity, specific volume, and instrumental color 24 h after production. Shelf‐life determination included analyses of fungal count (CFU/g), instrumental texture, and moisture content. For tests with EO application in the dough, analyses were conducted after 1, 4, and 7 days of storage, while for samples subjected to spraying, analyses were performed after 1, 4, 7, 10, 13, 16, 19, and 22 days of storage.

Water activity determination was done by direct measurement using the Aqualab hygrometer (Decagon, 4TE Duo, Pullman, USA). Specific volume was determined using the displaced seed method through method 10‐05.01 [38]. Instrumental crumb color was assessed using the 14‐22.01 method [38] on a spectrophotometric colorimeter (Konica Minolta, CM5, Chiyoda, JAP). Illuminant D65, a 10° angle for the observer, and the CIELAB color system were established. The moisture content of the bread was determined by oven drying (Tecnal, TE‐394/1, Piracicaba, BRA) at 130 ± 2°C, according to method 44‐15.02 [38]. The instrumental texture of the bread was determined using a Stable Micro Systems texture analyzer (TAX.XT Plus, Godalming, ENG), through method 74‐09.01 [38], using a cylindrical probe P/36 and HDP/90 platform to assess crumb firmness. Test parameters included pre‐test, test, and post‐test speeds of 1 mm/s, 1 mm/s, and 10.0 mm/s, respectively, a detection threshold of 0.049 N, and a compression distance of 40%, using two slices of 12 mm thickness each (Venancio, FPV129, Venâncio Aires, BRA). The texture analysis conditions were optimized through preliminary trials to ensure accurate and reliable assessment. Microbiological evaluation of the samples was done using the standard method for mold and yeast count (method no. 42.50‐01) [38]. Results were expressed in colony‐forming units per gram of sample (CFU/g). All analyses were performed in triplicate, except for crumb firmness, for which eight repetitions were conducted.

## 5 Statistical Analysis

The data obtained in the experimental planning were evaluated through response surface methodology to calculate the regression coefficient and analysis of variance (ANOVA) at a 10% significance level, using Statistica 13.5 (TIBCO Software Inc., 2019, Palo Alto, CA, USA). The minimum coefficient of determination (R^2^) from ANOVA was 0,80 [43, 47]. The mathematical models were presented for the coded values of the independent variables x_1_ (cinnamon EO), x_2_ (clove EO), and x_3_ (bay EO). The optimized point was determined according to the methodology proposed by Derringer and Suich [48].

The results for bread characterization were evaluated using linear regression analysis, with mathematical models considered valid when they exhibited a regression coefficient ≥ 0.80, with 95% confidence. For the microbiological analysis results, ANOVA and Tukey's test (*p* < 0.05) were conducted.

## Author Contributions

**Conceptualization**: Franciele Maria Pelissari and Marcio Schmiele; **Data curation**: Franciele Maria Pelissari and Marcio Schmiele; **Formal analysis**: Mariana Silveira, Karine Guimarães Moreira, Míriam Aparecida de Aguilar Santos, Irene Andressa, Mateus Alves de Araújo, and Nathalia Neves; **Funding acquisition**: Marcio Schmiele; **Investigation**: Mariana Silveira and Míriam Aparecida de Aguilar Santos; **Methodology**: Mariana Silveira, Karine Guimarães Moreira, and Nathalia Neves, Franciele Maria Pelissari and Marcio Schmiele; **Project administration**: Franciele Maria Pelissari and Marcio Schmiele; **Supervision**: Franciele Maria Pelissari and Marcio Schmiele; **Visualization**: Mariana Silveira, Karine Guimarães Moreira, and Míriam Aparecida de Aguilar Santos, Irene Andressa, Mateus Alves de Araújo, Nathalia Neves, Franciele Maria Pelissari and Marcio Schmiele; Writing—original draft, Mariana Silveira; Writing—review & editing, Irene Andressa, Nathalia Neves and Marcio Schmiele.

## Conflicts of Interest

The authors declare no conflicts of interest.

## Funding

The authors thank the Federal University of the Jequitinhonha and Mucuri Valleys (UFVJM) and the Institute of Science and Technology (ICT) for institutional and financial support (Process 23086.001699/2022‐41), the Coordination for the Improvement of Higher Education Personnel (CAPES) for financial support (code 001), and the National Council for Scientific and Technological Development (CNPq) (process #424938/2016‐2) and research scholarship (#312759/2025‐8).

## Supporting information




**Supporting Files 1**: cbdv70612‐sup‐0001‐SuppMat.docx

## Data Availability

The authors have nothing to report.
