# Supplementary material for: Enhancing Wholemeal Bread Shelf Life Using Optimized Mixtures of Cinnamon, Clove, and Bay Leaf Essential Oils
Source: Chem Biodivers. 2025 Oct 30;23(1):e01988. doi: 10.1002/cbdv.202501988 (PMC12761357; doi:10.1002/cbdv.202501988)
Supplement: Supplementary file 1 — Supporting Files 1: cbdv70612‐sup‐0001‐SuppMat.docx [file CBDV-23-e01988-s001.docx]

Supplementary Material

**Table S1** – Diameter of inhibition zone (cm) by disk diffusion test using different concentrations of cinnamon, clove and bay laurel essential oils

| **Assay** | **Incubation time (days)** | | | | | | | |  |  |  |
| --- | --- | --- | --- | --- | --- | --- | --- | --- | --- | --- | --- |
|  | **1** | **2** | **3** | **4** | **5** | **6** | **7** | **8** | **1/5*** | **4/5*** | **1/8*** |
| 1 | 3.0±0.42 | 2.3±0.39 | 2.1±0.25 | 2.1±0.25 | 1.2±0.08 | 1.1±0.07 | 1.1±0.07 | 1.1±0.08 | 60.00±0.43 | 42.86±0.26 | 63.30±0.43 |
| 2 | 2.3±0.22 | 2.0±0.24 | 2.0±0.24 | 2.0±0.23 | 1.9±0.25 | 1.9±0.27 | 1.9±0.27 | 1.9±0.24 | 17.39±0.33 | 5.00±0.34 | 17.39±0.33 |
| 3 | 2.5±0.24 | 2.5±0.30 | 2.5±0.32 | 2.5±0.32 | 2.5±0.32 | 2.3±0.28 | 2.3±0.28 | 2.2±0.23 | 0.00±0.41 | 0.00±0.46 | 12.00±0.34 |
| 4 | 2.3±0.16 | 2.3±0.10 | 2.3±0.10 | 2.3±0.10 | 2.3±0.09 | 2.2±0.07 | 2.2±0.07 | 2.2±0.11 | 2.13±0.19 | 0.00±0.14 | 6.38±0.20 |
| 5 | 2.1±0.11 | 2.0±0.14 | 1.9±0.16 | 1.9±0.16 | 1.2±0.09 | 0.6±0.23 | 0.6±0.25 | 0.5±0.28 | 42.86±0.14 | 36.84±0.19 | 76.19±0.30 |
| 6 | 2.5±0.09 | 2.5±0.09 | 2.4±0.17 | 2.4±0.16 | 2.2±0.15 | 2.0±0.17 | 2.0±0.17 | 2.0±0.20 | 12.00±0.18 | 8.33±0.22 | 20.00±0.22 |
| 7 | 2.6±0.24 | 2.6±0.29 | 2.4±0.32 | 2.4±0.32 | 2.4±0.32 | 2.3±0.28 | 2.3±0.28 | 2.3±0.23 | 7.69±0.41 | 0.00±0.46 | 11.54±0.34 |
| 8 | 2.6±0.25 | 2.5±0.15 | 2.5±0.07 | 2.5±0.07 | 2.5±0.05 | 2.5±0.09 | 2.5±0.09 | 2.5±0.08 | 3.85±0.26 | 0.00±0.08 | 3.85±0.27 |
| 9 | 2.5±0.15 | 2.3±0.15 | 2.3±0.15 | 2.2±0.10 | 2.0±0.09 | 1.9±0.15 | 1.9±0.08 | 1.9±0.15 | 20.00±0.18 | 9.09±0.14 | 24.00±0.22 |
| 10 | 2.7±0.08 | 2.6±0.05 | 2.6±0.05 | 2.6±0.05 | 2.6±0.05 | 2.6±0.08 | 2.6±0.07 | 2.6±0.07 | 3.70±0.10 | 0.00±0.07 | 3.70±0.11 |
| 11 | 1.9±0.07 | 1.9±0.07 | 1.9±0.07 | 1.8±0.07 | 1.4±0.09 | 1.3±0.05 | 1.3±0.15 | 1.3±0.13 | 26.32±0.12 | 22.22±0.12 | 31.58±0.15 |
| 12 | 2.5±0.12 | 2.5±0.12 | 2.5±0.12 | 2.5±0.13 | 2.5±0.11 | 2.5±0.11 | 2.4±0.11 | 2.4±0.18 | 0.00±0.17 | 0.00±0.17 | 4.00±0.22 |
| 13 | 2.1±0.49 | 2.1±0.41 | 2.1±0.41 | 2.1±0.25 | 2.1±0.10 | 2.0±0.33 | 2.0±0.33 | 2.0±0.11 | 0.00±0.50 | 0.00±0.27 | 4.76±0.50 |
| 14 | 2.4±0.34 | 2.3±0.27 | 2.3±0.27 | 2.3±0.17 | 2.2±0.19 | 2.2±0.21 | 2.2±0.21 | 2.2±0.07 | 8.33±0.39 | 4.35±0.26 | 8.33±0.35 |
| 15 | 2.2±0.11 | 2.1±0.18 | 2.1±0.18 | 2.1±0.18 | 2.0±0.12 | 2.0±0.16 | 2.0±0.16 | 2.0±0.14 | 9.09±0.17 | 4.76±0.22 | 9.09±0.18 |
| 16 | 2.2±0.11 | 2.1±0.09 | 2.1±0.09 | 2.0±0.05 | 2.0±0.05 | 1.9±0.10 | 1.9±0.10 | 1.9±0.10 | 9.09±0.12 | 0.00±0.07 | 13.64±0.15 |
| 17 | 2.2±0.26 | 2.2±0.25 | 2.2±0.25 | 2.1±0.18 | 2.1±0.18 | 2.0±0.17 | 2.0±0.18 | 2.0±0.20 | 4.55±0.32 | 0.00±0.26 | 9.09±0.18 |
| 18 | 2.2±0.33 | 2.2±0.29 | 2.2±0.29 | 2.1±0.18 | 2.0±0.18 | 1.9±0.16 | 1.9±0.16 | 1.9±0.16 | 9.09±0.38 | 4.76±0.26 | 13.64±0.15 |
| The mean of three replicates ± Standard Deviation (SD) of the population; * The Percentage decrease of inhibition halo (the Mean difference between days of storage ± SD due to the propagation of error). | | | | | | | | | | | |

Table S2 - Mathematical models of dependent variables that are statistically significant (p<0,10), for the proportions of essential oils in *in vitro* assays

| **Dependent variable (inhibition halo through the incubation days)** | **Mathematical model** |
| --- | --- |
| 2 (cm) | 2.19+0.16x_2_+0.07x_3_+0.10x_1_^2^**-**0.05x_1_x_2_–0.12x_1_x_3_ |
| 3 (cm) | 2.17+0.06x_1_+0.17x_2_+0.05x_3_+0.10x_1_^2^**-**0.06x_1_x_2_ +0.11x_1_x_3_ |
| 4 (cm) | 2.10+0.07x_1_+0.18x_2_+0.05x_3_+0.11x_1_^2^+ 0.04x_3_^2^–0.06x_1_x_2_+0.11x_1_x_3_ |
| 5 (cm) | 2.05+0.19x_1_+0.37x_2_+0.04x_3_+0.07x_1_^2^–0.07x_2_^2^–0.22x_1_x_2_ +0.07x_1_x_3_ |
| 6 (cm) | 1.96+0.25x_1_+0.42x_2_+0.06x_1_^2^–0.07x_2_^2^**-**0.26x_1_x_2_+0.11x_1_x_3_+0.09x_2_x_3_ |
| 7 (cm) | 1.92+0.26x_1_+0.41x_2_–0.26x_1_x_2_ |
| 8 (cm) | 1.96+0.27x_1_+0.41x_2_+0.05x_1_^2^–0.09x_2_^2^–0.26x_1_x_2_+0.11x_1_x_3_+0.11x_2_x_3_ |
| 1/5 (%) | 8.05–7.52x_1_–11.93x_2_+3.11x_1_^2^+3.57x_2_^2^+8.97x_1_x_2_+3.99x_2_x_3_ |
| 4/5 (%) | 4.83–5.98x_1_–9.55x_2_+3.75x_2_^2^+8.29x_1_x_2_ |
| 1/8 (%) | 12.01-10.96x_1_–13.89x_2_+3.56x_1_^2^+4.96x_2_^2^+11.10x_1_x_2_–2.31x_2_x_3_ |
| x_1_: cinnamon essential oil; x_2_: clove essential oil and x_3_: bay essential oil; * Percentage decrease of inhibition halo | |

Table S3 - UFC/g of whole wheat bread with application of cinnamon, clove, and bay essential oils on the surface

| **Amostra** | **Time (days)** | | | | | | | |
| --- | --- | --- | --- | --- | --- | --- | --- | --- |
|  | **1** | **4** | **7** | **10** | **13** | **16** | **19** | **22** |
| SS | nd | nd | 1.60±0.29x10^2^ | 4.66±0.47x10^2 a^ | 7.27±1.87x10^3 a^ | 1.60±0.29x10^4 a^ | 6.00±2.16x10^4 a^ | 3.00±0.22x10^5 a^ |
| SC | nd | nd | nd | nd | nd | 7.67±2.05x10^1 b^ | 8.33±1.24x10^2 c^ | 3.67±0.20x10^3 c^ |
| S3 | nd | nd | nd | 3.33±0.05x10^1 b^ | 2.60±0.21x10^2 b^ | 4.03±0.45x10^3 b^ | 4.03±0.45x10^4 ab^ | 1.30±0.22x10^5 b^ |
| S6 | nd | nd | nd | 5.67±0.94x10^1 b^ | 1.96±0.20x10^2 b^ | 2.30±0.21x10^3 b^ | 2.30±0.22x10^4 bc^ | 9.00±0.82x10^4 b^ |
| S9 | nd | nd | nd | nd | nd | 1.30±0.43x10^2 b^ | 6.07±0.25x10^3 c^ | 3.37±0.86x10^4 c^ |
| S12 | nd | nd | nd | nd | nd | nd | 2.67±0.47x10^2 c^ | 3.00±0.82x10^3 c^ |
| Mean of three replicates ± standard deviation; nd - not detected or value below the detection limit (10 CFU/g) | | | | | | | | |
